# Validating LENT score in malignant pleural effusion

**DOI:** 10.2144/fsoa-2023-0168

**Published:** 2024-05-20

**Authors:** Alia Alawneh, Fadi Abu Farsakh, Mahmoud Smadi, Shaher Samrah

**Affiliations:** 1 Jordan University of Science & Technology, Faculty of Medicine, Internal Medicine Department, 3030, Ar-Ramtha, Jordan; 2 King Hussein Cancer Center, Palliative Medicine Department, Queen Rania St 202, Amman, Jordan; 3 Jordan University of Science & Technology, Faculty of Science, Mathematics Department, 3030, Ar-Ramtha, Jordan; 4 Jordan University of Science & Technology, Faculty of Medicine, Internal Medicine Department, Pulmonary & Critical Care Division, 3030, Ar-Ramtha, Jordan

**Keywords:** LENT score, malignancy, pleural effusion, prognosis, validation

## Abstract

**Aim:** LENT score was developed to predict survival in malignant pleural effusion (MPE), this study aims to validate this score. **Objectives:** Validate LENT Prognostic Score for MPE and explore survival in these patients. **Methods:** Retrospective analysis of 202 patients who had MPE and received drainage between January 2013 and June 2015. **Results:** Median survival was 2.98 months. Patients were classified according to LENT score as low, moderate and high-risk groups: 5 (4.2%), 61 (50.8%), and 54 (45%), respectively. Kaplan–Meier curve showed median survival for each group: 9.41, 5.36 and 0.56 months, respectively, p-values <0.001. AUC for 1, 3 and 6 months: 0.741, 0.781, 0.790, respectively, p-values <0.001. **Conclusion:** LENT score is valid for predicting survival in patients with MPE.

## Abstract (plain language summary)

LENT score was developed to predict survival in malignant pleural effusion, the aim of this study is to validate LENT score as prognostic model in a different temporal and geographical area. In addition, this study explores survival of patients with MPE.

Study results showed median survival was 2.98 months. LENT score proved valid in predicting survival in patients with MPE.

In conclusion, LENT score can be used in clinical decision making and to help patients make well informed decisions.

Malignant pleural effusion (MPE) affects 15% of patients with cancer, most commonly lung cancer patients, followed by breast cancer, lymphoma, gynaecological malignancies and malignant mesothelioma. The annual incidence of MPE is estimated to be 150,000 cases [1]. Median survival following a diagnosis of MPE ranges from 3 to 12 months, depending on the stage of malignancy, type of malignancy and treatments received. Studies have shown that shortest survival time was in patients with MPE secondary to lung cancer, and longest in patients with ovarian cancer [2]. MPE can cause significant morbidity and affects the quality of life by causing distressing symptoms including dyspnoea, orthopnoea, cough, and chest pain. The severity of these symptoms depends on the amount of the effusion and the patients' cardiopulmonary status [3].

There are different options to treat MPE, including observation, intermittent thoracentesis, indwelling pleural catheter insertion (IPCI), intercostal tube drainage, thoracoscopy and pleurodesis. Treatments may cause morbidity, necessitate hospitalization, in addition to financial burden. Therefore, patient's prognosis plays an important role in selecting the most appropriate treatment that aligns with the patients' goals of care and minimizes discomfort and inconvenience.

Over the past years, many prognostic tools were developed to predict prognosis of patients with MPE, including LENT, modified LENT, SELECT and PROMISE scores. Currently, the most established and validated method to predict survival in patients with MPE is LENT score (L – LDH level; E – ECOG; N – neutrophil-to-lymphocyte ration; T – tumor type), and it was found to be more helpful than depending solely on the performance status (ECOG or Palliative Performance Scale) [4].

LENT score is a prognostic score in patients with MPE that was developed and externally validated using three large international cohorts from the UK, the Netherlands and Australia [4]. For prognostic models, there should be an ongoing process of model validation which can be geographical or temporal [5]. In this study, we are validating the LENT score among patients treated at King Hussein Cancer Centre in Amman, Jordan.

## Methods

### Objectives

Primary objective was to externally validate LENT score as prognostic score in patients with MPE. Secondary objective was to explore overall survival in patients with MPE who received IPCI.

### Patients & setting

This is a retrospective analysis of cancer patients treated at King Hussein Cancer Centre in Amman, Jordan who received IPCI for MPE between January 2013 and June 2015. Inclusion criteria was for any patient who was diagnosed with MPE and received IPCI for the first time. We used the same diagnostic criteria used by Clive AO *et al.* for diagnosing MPE [4]. MPE was diagnosed using cytopathology and using clinical diagnosis including symptoms and images when cytopathology was not available. These were included regardless whether they received treatment or not and with disregard for the time of MPE diagnosis during the cancer trajectory. Patients were followed until death or time of data analyses; median follow-up time was 28.5 months (range; 13.4–62.3 months). This study was carried in compliance with Declaration of Helsinki and was approved by the Institutional Review Board at King Hussein Cancer Centre.

### Model to validate

LENT score constitutes of serum lactate dehydrogenase (LDH), Eastern Cooperative Oncology Group (ECOG) Performance Score, blood neutrophil/lymphocyte ratio, and tumor type. The score ranges from 0–7. The patients are divided into low-risk (score 0–1), moderate-risk (score 2–4) and high-risk (score 5–7) prognostic groups [4].

### Statistical analysis

Descriptive data were reported as absolute numbers and percentages or mean and standard deviations as appropriate. Some visual examination data are also presented.

The LENT prognostic-score is implemented to measure time from (IPCI) insertion to death. Kaplan–Meier method was used to assess the overall survival rates and to develop survival curves. Overall survival was calculated from the date of IPCI insertion to death. The Kaplan–Meier method of estimating the survival function is a bivariate nonparametric statistical method based on estimating conditional probabilities at each time point when an event occurs in the presence of censored cases and taking the product limit of those probabilities to estimate the survival rate at each point in time. Kaplan–Meier curves are often employed in medicine to test the difference between treatment groups such as mortality and recurrence.

The prognostic utility of LENT score was assessed to predict mortality at 1 month, 3 months and 6 months by determining the area under the receiver operating characteristic curve (AUC) with 95% CI, standard errors, and p-values. The c-statistic is a measure of how well the model can discriminate between observations at different levels of the outcome. This is similar to the area under the receiver-operating characteristic curve. The minimum value of c that can take values ranges between 0.5 and 1.0. Hosmer and Lemeshow [6] considered c values between 0.7 and 0.8 to be acceptable for discrimination, values between 0.8 and 0.9 indicates excellent discrimination, and values above or equal of 0.9 as outstanding discrimination [6].

Binary logistic model was fitted where the LENT score was considered as independent or predictor variable, and the 1 month, or 3 months, or 6 months' mortality as an outcome or dependent variable. Nagelkerke R2 statistic values, odds ratio (OR) and predicted death probabilities for one month, 3 months and 6 months' mortality were reported. IBM Statistical analysis software SPSS version 23 was used to perform the data analysis.

### Sample size

Our sample included patients who received IPCI for MPE between the time periods of January 2013 until June 2015. To verify that our sample size is adequate, we used the rule of ten events for each potential prognostic variable [7]. As LENT score constitutes of four variables, according to the previous rule, 40 patients are needed at least to have adequate sample size.

## Results

**Figure 1.**
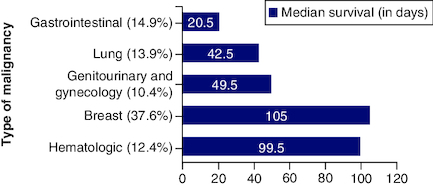
Median survival in days according to type of malignancy.

In the study, 202 patients were identified; among those 131 (64.9%) were females, ranging from 18 to 85 years of age with median age of 54 years. As shown in Figure 1, breast cancer was the most common cancer type (37.6%), followed by lung cancer (13.9%). In all cases, no major life-threatening complications occurred due to catheter insertion. However, 21.2% (n = 43) of catheters were removed because of complete fluid drainage (11 patients), loculated fluid (28 patients), or plugged catheter (four patients). The mean catheter survival was 95.5 days (ranging from 1–746 days), Table 1 shows baseline patients' characteristics.

**Table 1.** Baseline characteristics.

Variable	Absolute n (%)
Age (years)	Mean 54.15 years, range 18–85 years
Gender Male Female	71 (35.1%) 131 (64.9%)
Laterality Left Right Bilateral	89 (44.05%) 85 (42.07%) 28 (13.86%)
Smoking status Smokers Non-smokers Missing	20 (9.9%) 72 (35.64%) 110 (54.45%)
Mode of diagnosis Pleural cytology, flow cytometry or pleural biopsy Otherwise unexplained effusion with confirmed malignancy elsewhere	64 (31.6%) 138 (68.3%)

Upon calculating the LENT score, 82 patients were excluded from the analysis due to incomplete data needed to calculate the LENT score. Thus, 120 patients' data was analysed. At the time of the first pleural drainage, Patients were categorized according to their LENT score into low (LENT 0–1), moderate (LENT 2–4) and high (LENT 5–7) risk groups. The low-risk group comprised of 5 (4.2%) patients, the moderate-risk group comprised of 61 (50.8%) patients and the high-risk group comprised of 54 (45%) patients. 108 patients died by the end of the observation period and 12 were censored, the overall median survival was 62 days (2.06 months), Table 2.

**Table 2.** Distribution of LENT categories and median survival.

LENT Category	Count	Percent	Median survival (months)
Low risk	5	4.2%	9.41
Moderate risk	61	50.8%	5.36
High risk	54	45.0%	0.55
Overall median survival			2.06

**Figure 2.**
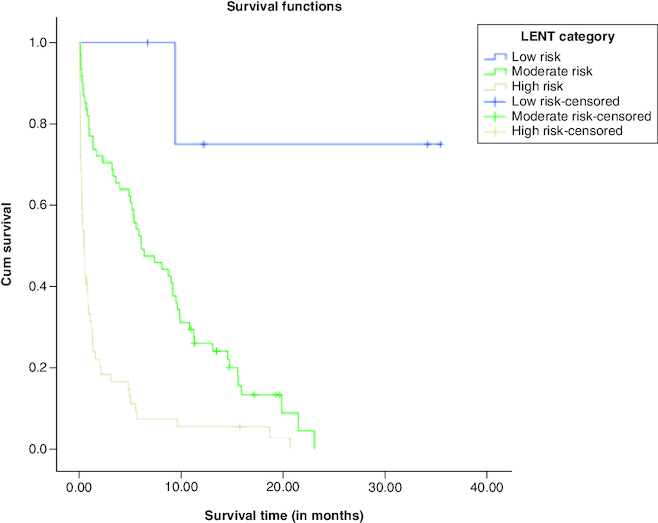
Survival functions according to LENT score (low, moderate and high) risk.

The K-M survival curves for LENT scores are shown in Figure 2. It is noted that the cumulative survival function for high risk LENT score decays sharply, whereas the cumulative survival function for moderate risk LENT score decays slowly. The cumulative survival function for low risk LENT score involves one step and then becomes in a constant. In addition, the survival functions of the three LENT risk scores (low risk, moderate risk and high risk) were compared using log rank, Breslow and Tarone-Ware statistical tests. All of the statistical tests revealed p-values <0.001, indicating that the survival curves for low risk score, moderate risk score, and high risk score are statistically significant. Pairwise comparisons using log rank, Breslow and Tarone-Ware indicated that survival curves for low risk, moderate risk and high risk are statistically significant.

**Figure 3.**
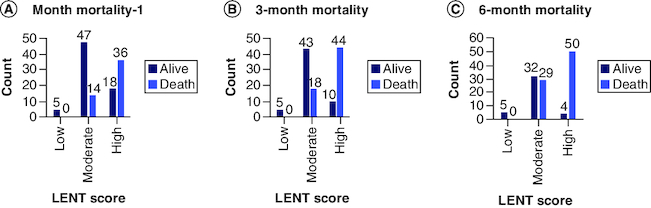
Bar chart of mortality versus LENT score (low, moderate, high) risk. **(A)** 1-month mortality, **(B)** 3-month mortality **(C)** 6-month mortality.

Clustered bar chart of LENT risk score (low, moderate and high) versus 1 month, 3 months and 6 months' mortality (death, alive) were shown in Figures 3A, B & C, respectively. It is noted that there is a remarkable increase in the frequency of death cases compared with alive cases with high LENT risk score for all 1 month, 3 months and 6 months' mortality.

Survival data can also be presented in terms of median survival time, which is the time point at which the cumulative survival drops below 50%. Patients with low risk LENT score with a median survival of 9.41 months, revealed 100% survival at 1, 3 and 6 months' mortality. Those with a moderate risk LENT score with a median survival of 5.36 months, their survival at 1, 3 and 6 months were 81.2%, 75% and 23%, respectively. Those with a high risk LENT score with a median survival of only 0.55 month, their survivals at 1, 3 and 6 months' mortality, were 30.7%, 21.2% and 9.6%, respectively.

**Figure 4.**
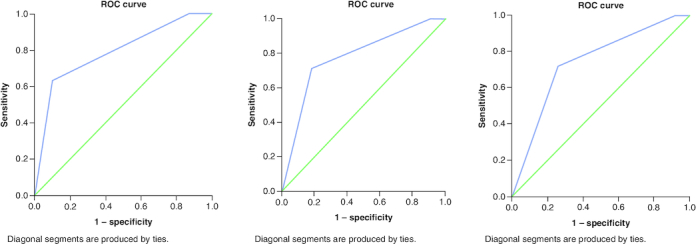
ROC curve for LENT score for the outcome of mortality. At **(A)** 1-month mortality **(B)** 3-month mortality **(C)** 6-month time periods. AUC was 0.74, 0.78 and 0.79, respectively. All p-values <0.001.

ROC curve analysis was conducted for LENT risk score (low, moderate, and high) as a test variable and 1 month, 3 months and 6 months' mortality as a state variable, Figure 4. AUC for 1, 3 and 6 months: 0.741, 0.781, 0.790, respectively, p-values <0.001. All results are acceptable for discrimination.

Binary logistic model was fitted for LENT risk score as a predictor, and 1 month, 3 months and 6 months' mortality (death, alive) as outcome variable. For all cases, the results showed that LENT risk score was a significant predictor for mortality for the three-time mortality periods, with all p-values <0.001. The Nagelkerke R2 values were found to be 28.1%, 37.9%, and 39.7% for 1 month, 3 months and 6 months' mortality periods, respectively.

The OR (odds ratio) values were found to be 7.078, 11.051 and 15.401 for 1 month, 3 months and 6 months' mortality, respectively. The predicted death probability for the 1 month, 3 months and 6 months' mortality periods were found to be 0.039, 0.036 and 0.054 for low LENT risk score, 0.223, 0.289, and 0.467 for moderate LENT risk score, and 0.67, 0.818 and 0.930 for high LENT risk score, respectively. The predicted death probabilities for LENT scores for the 1 month, 3 months and 6 months' mortality periods are shown in Table 3.

**Table 3.** Predicted death probability for LENT scores; 1 month, 3 months and 6 months mortality groups.

Groups	Low LENT risk score	Moderate LENT risk score	High LENT risk score
1 months	0.039	0.223	0.670
3 months	0.036	0.289	0.818
6 months	0.054	0.467	0.930

## Discussion

This study was designed to validate LENT score in a different geographical area than the development study. Our results validate LENT score as a useful tool to predict survival in patients with MPE receiving IPC. Predicting survival in patients with MPE provides valuable prognostic information to the patients, caregivers and healthcare providers to enable a proper discussion of goals of care that aligns with patient's values, preferences and expected prognosis, and to set a treatment plan that focus on the relief of dyspnoea, maintaining independency as much possible, and avoidance of inpatient care.

IPC can be used to treat first episode of MPE and is usually the first step in management of MPE. However, in patients with very poor prognosis, observation and medical management using pharmacological and non-pharmacological approaches are more beneficial, such as oxygen (if the patient is hypoxic), use of hand-held fan, diuretics and opioids to help in treating dyspnoea.

LENT score was developed and validated by Clive *et al.* [4] to predict survival in patients with MPE. The primary tumor types were lung cancer, breast cancer and mesothelioma. In our sample, breast cancer was the commonest tumor type, followed by lung cancer and colon cancer. Our study represented a different sample compared with the development study in regards to age and gender; the median age in our study was 54 years compared with 66 in the development study. The majority in our sample were females 64.9%, in contrast to the development study where they had 53.6% of males. Yet, our study showed similar results to the original study regarding median survival in low-risk, moderate-risk and high-risk LENT scores.

LENT score can be simply collected and calculated; a high pleural fluid LDH levels reflect localized, acute inflammation, necrosis and cell death within the pleural cavity, it is not routinely done for all our patients, but it is a cheap test that can give a better prognostic value. ECOG is a very helpful and simple tool to predict prognosis in patients with advanced cancer, LENT score showed to be significantly better than ECOG alone in predicting survival [8]. Neutrophil-to-lymphocyte ratio (NLR) can be simply calculated from the routine complete blood count (CBC); a routine and universally available test, its significance in predicting survival was supported in different studies [9,10], and was found to be a predictor of a reduced survival independent of age, sex and tumor type. The tumor type plays an important factor in prognostication as well. In our sample, we had one patient with mesothelioma with a survival of 32 days post IPCI. Whereas the average survival rate for hematologic malignancies was 123.85 days. The tumor type is a challenging factor in predicting prognosis. Sensitivity to chemotherapy is variable among patients, and it is an important factor in affecting survival. Even in patients with lung cancer, there are different histological types that have different responses to treatment, and new emerging treatment options, including biological treatments, can affect the overall prognosis. In addition, availability and improvements in palliative care treatments in metastatic lung cancer increase the survival and improve the quality of life of those patients [11].

It is crucial to determine the prognosis of the patient and the impact of MPE on quality of life to set the treatment plan accordingly. While the majority of patients with MPE will experience symptoms affecting their quality of life at some point of their illness, some may remain asymptomatic and can be observed. Dyspnoea is the commonest symptom of MPE in 96% of symptomatic patients; chest pain comes second (57.5%) and cough third (44%) [12].

IPCI is the most appropriate treatment, yet it should be individualized after considering patient's preferences, performance status, and the setting of the care (inpatient vs home). It is generally a safe procedure, especially when performed under ultrasound guidance. It is considered to be a minimally invasive intervention and appropriate for patients who prefer outpatient management. Re-expansion pulmonary oedema can occur infrequently after the removal of more than 1.5 l of fluids (less than 0.5% as per) [13]. On the other hand, Wahidi *et al.* [14] concluded that daily drainage of pleural fluid via IPC led to a higher rate of autopleurodesis.

The complications of IPC include bleeding during the procedure of insertion, thus checking the coagulation profile of the patients is mandated. Other complications include catheter blockage, catheter fracture or removal, tract metastases and infections, which can be at the point of insertion, along the catheter tract, or within pleural space [15]. In our cohort, no major life threatening complications occurred due to catheter insertion. In 43 cases (21.2%) of our sample, the catheter was removed mainly due to blockage of the catheter, presence of loculated effusion or autopleurodesis. International randomized ratio (INR) and complete blood count (CBC) were checked 24–48 h prior to the procedure.

Talc pleurodesis is an alternative option to treat recurrent MPE, and can be used in cases of IPC failure; it is performed by installation of talc poudrage to the intrapleural space. This procedure is suitable for patients who have a reasonable life expectancy (more than 3–6 months), or who prefer a more definitive and rapid procedure that minimizes future interventions, of note, patients with nonexpandable lungs are not candidates for chemical pleurodesis. Pleurodesis was not shown to result in improved dyspnoea symptom nor hospitalization days than does indwelling pleural catheters [15,16]. Other procedures that can be rarely used include pleuroperitoneal shunting and pleurectomy.

The significance of the LENT score should also be communicated to oncological decision-making. We are in the era of artificial intelligence and more complex models with better performance may develop [17].

### Limitations

This study is retrospective and did not evaluate the subjective improvement in patients' symptoms after IPC placement or the impact of the procedure on the quality of life of these patients.

## Conclusion

MPE has a poor prognosis, and therapy should be determined by life expectancy in order to provide patients with treatment options that maximize the control of their symptoms and quality of life.

## References

[1] Neragi-Miandoab S. Malignant pleural effusion, current and evolving approaches for its diagnosis and management. Lung cancer (Amsterdam, Netherlands). 54(1), 1–9 (2006). 16893591 10.1016/j.lungcan.2006.04.016

[2] Roberts ME, Neville E, Berrisford RG, Antunes G, Ali NJ. Management of a malignant pleural effusion: British Thoracic Society pleural disease guideline 2010. Thorax 65(Suppl. 2), ii32–ii40 (2010). 20696691 10.1136/thx.2010.136994

[3] Lorenzo MJ, Modesto M, Pérez J et al. Quality-of-Life assessment in malignant pleural effusion treated with indwelling pleural catheter: a prospective study. Palliat. Med. 28(4), 326–334 (2014). 24523284 10.1177/0269216314521851

[4] Clive AO, Kahan BC, Hooper CE et al. Predicting survival in malignant pleural effusion: development and validation of the LENT prognostic score. Thorax 69(12), 1098–1104 (2014). 25100651 10.1136/thoraxjnl-2014-205285 PMC4251306

[5] Steyerberg EW, Moons KG, van der Windt DA et al. Prognosis Research Strategy (PROGRESS) 3: prognostic model research. PLOS Med. 10(2), e1001381 (2013). 23393430 10.1371/journal.pmed.1001381 PMC3564751

[6] Hosmer DW Jr, Lemeshow S, Sturdivant RX. Applied logistic regression. John Wiley & Sons, NJ, USA (2013).

[7] Altman DG, Vergouwe Y, Royston P, Moons KG. Prognosis and prognostic research: validating a prognostic model. BMJ 338, b605 (2009). 19477892 10.1136/bmj.b605

[8] Jang RW, Caraiscos VB, Swami N et al. Simple prognostic model for patients with advanced cancer based on performance status. J. Oncol. Pract. 10(5), e335–341 (2014). 25118208 10.1200/JOP.2014.001457

[9] Kao SC, Pavlakis N, Harvie R et al. High blood neutrophil-to-lymphocyte ratio is an indicator of poor prognosis in malignant mesothelioma patients undergoing systemic therapy. Clin. Cancer Res. 16(23), 5805–5813 (2010). 20956618 10.1158/1078-0432.CCR-10-2245

[10] Anevlavis S, Kouliatsis G, Sotiriou I et al. Prognostic factors in patients presenting with pleural effusion revealing malignancy. Respiration 87(4), 311–316 (2014). 24457947 10.1159/000356764

[11] Temel JS, Greer JA, Muzikansky A et al. Early palliative care for patients with metastatic non-small-cell lung cancer. N. Engl. J. Med. 363(8), 733–742 (2010). 20818875 10.1056/NEJMoa1000678

[12] Martínez-Moragón E, Aparicio J, Sanchis J, Menéndez R, Cruz Rogado M, Sanchis F. Malignant pleural effusion: prognostic factors for survival and response to chemical pleurodesis in a series of 120 cases. Respiration 65(2), 108–113 (1998). 9580921 10.1159/000029240

[13] Feller-Kopman D, Berkowitz D, Boiselle P, Ernst A. Large-volume thoracentesis and the risk of reexpansion pulmonary edema. Ann. Thoraci. Surg. 84(5), 1656–1661 (2007). 10.1016/j.athoracsur.2007.06.038 17954079

[14] Wahidi MM, Reddy C, Yarmus L et al. Randomized Trial of Pleural Fluid Drainage Frequency in Patients with Malignant Pleural Effusions. The ASAP Trial. Am. J. Respir. Crit. Care Med. 195(8), 1050–1057 (2017). 27898215 10.1164/rccm.201607-1404OC

[15] Davies HE, Mishra EK, Kahan BC et al. Effect of an indwelling pleural catheter vs chest tube and talc pleurodesis for relieving dyspnea in patients with malignant pleural effusion: the TIME2 randomized controlled trial. JAMA 307(22), 2383–2389 (2012). 22610520 10.1001/jama.2012.5535

[16] Thomas R, Fysh ETH, Smith NA et al. Effect of an Indwelling Pleural Catheter vs Talc Pleurodesis on Hospitalization Days in Patients With Malignant Pleural Effusion: The AMPLE Randomized Clinical Trial. JAMA 318(19), 1903–1912 (2017). 29164255 10.1001/jama.2017.17426 PMC5820726

[17] Loureiro H, Becker T, Bauer-Mehren A, Ahmidi N, Weberpals J. Artificial Intelligence for Prognostic Scores in Oncology: a Benchmarking Study. Front. Artif. Intel. 4, 625573 (2021). 10.3389/frai.2021.625573 PMC8086599 33937744

